# Superoxide Dismutase Mimetic Avasopasem Manganese Enhances Radiation Therapy Effectiveness in Soft Tissue Sarcomas and Accelerates Wound Healing

**DOI:** 10.3390/antiox13050587

**Published:** 2024-05-10

**Authors:** Amira Zaher, Kranti A. Mapuskar, Michael S. Petronek, Munir R. Tanas, Alexandra L. Isaacson, Rebecca D. Dodd, Mohammed Milhem, Muhammad Furqan, Douglas R. Spitz, Benjamin J. Miller, Robert A. Beardsley, Bryan G. Allen

**Affiliations:** 1 Department of Radiation Oncology, The University of Iowa, Iowa City, IA 52242, USA; amira-zaher@uiowa.edu (A.Z.); krantiashok-mapuskar@uiowa.edu (K.A.M.); michael-petronek@uiowa.edu (M.S.P.); douglas-spitz@uiowa.edu (D.R.S.); 2 Department of Pathology, The University of Iowa, Iowa City, IA 52242, USA; munir-tanas@uiowa.edu (M.R.T.); isaacsa2@ccf.org (A.L.I.); 3 Department of Pathology, The Cleveland Clinic, Cleveland, OH 44195, USA; 4 Department of Internal Medicine, Division of Hematology and Oncology, The University of Iowa, Iowa City, IA 52242, USA; rebecca-dodd@uiowa.edu (R.D.D.); mohammed-milhem@uiowa.edu (M.M.); muhammad-furqan@uiowa.edu (M.F.); 5 Department of Orthopedics and Rehabilitation, The University of Iowa, Iowa City, IA 52242, USA; benjamin-j-miller@uiowa.edu; 6 Galera Therapeutics Inc., 2 West Liberty Blvd., Suite 110, Malvern, PA 19355, USA; rbeardsley@galeratx.com

**Keywords:** soft tissue sarcoma, radiation therapy, wound healing, superoxide dismutase mimetic, avasopasem manganese

## Abstract

Soft tissue sarcomas (STSs) are mesenchymal malignant lesions that develop in soft tissues. Despite current treatments, including radiation therapy (RT) and surgery, STSs can be associated with poor patient outcomes and metastatic recurrences. Neoadjuvant radiation therapy (nRT), while effective, is often accompanied by severe postoperative wound healing complications due to damage to the surrounding normal tissues. Thus, there is a need to develop therapeutic approaches to reduce nRT toxicities. Avasopasem manganese (AVA) is a selective superoxide dismutase mimetic that protects against IR-induced oral mucositis and lung fibrosis. We tested the efficacy of AVA in enhancing RT in STSs and in promoting wound healing. Using colony formation assays and alkaline comet assays, we report that AVA selectively enhanced the STS (liposarcoma, fibrosarcoma, leiomyosarcoma, and MPNST) cellular response to radiation compared to normal dermal fibroblasts (NDFs). AVA is believed to selectively enhance radiation therapy by targeting differential hydrogen peroxide clearance in tumor cells compared to non-malignant cells. STS cells demonstrated increased catalase protein levels and activity compared to normal fibroblasts. Additionally, NDFs showed significantly higher levels of GPx1 activity compared to STSs. The depletion of glutathione using buthionine sulfoximine (BSO) sensitized the NDF cells to AVA, suggesting that GPx1 may, in part, facilitate the selective toxicity of AVA. Finally, AVA significantly accelerated wound closure in a murine model of wound healing post RT. Our data suggest that AVA may be a promising combination strategy for nRT therapy in STSs.

## 1. Introduction

Soft tissue sarcomas (STSs) comprise over 80 histotypes of heterogeneous mesenchymal malignancies that arise primarily in the extremities [1]. In the United States, 13,000 to 15,000 STS cases are diagnosed annually, with over 5000 deaths per year [2,3].

Although STSs represent only approximately 1% of all adult cancers, many STSs have high metastatic potential and poor patient outcomes [1,2,4,5] with 5-year survival rates between 50–60% [6]. STSs are less frequently studied and therapeutic advances can be slow [1,2,4,5]. 

The current management of STSs often involves radiation therapy (RT) combined with surgical excision. Other therapeutic agents, including chemotherapies, such as doxorubicin and ifosfamide, may also be utilized depending on tumor type, localization, stage, and the response to surgery and RT [7,8,9]. The timing of RT delivery in the treatment of STSs is critical for patient outcomes. Neoadjuvant radiation therapy (nRT) may improve tumor control compared to adjuvant RT [10,11]. However, nRT can cause severe wound healing complications following surgical excision in as many as one-third of patients with STSs [12]. Treatment with RT creates a highly oxidizing environment due to the increased generation of reactive oxygen species (ROS) [13]. Excess ROS can induce DNA, lipid, and protein damage, subsequently promoting inflammation and fibrosis [14,15]. With nRT, this results in the disruption and deceleration of the normal wound healing cascade following surgery, leading to complications, such as infection and necrosis, that may ultimately lead to amputation [12,16]. Thus, while nRT has substantial tumor control benefits, the consequential wound healing complications limit radiation doses in treating STSs. As a result, there is a significant need for selective therapeutic strategies which are capable of enhancing the efficacy of nRT and mitigating its damaging effects on surrounding normal tissues.

One of the primary ROS generated by RT is superoxide (O_2_^•−^) [13]. O_2_^•−^ can react with nitric oxide (NO) to form peroxynitrite (ONOO^−^), leading to protein damage [17]. Another fate of O_2_^•−^ is dismutation through the enzymatic activity of superoxide dismutase (SOD) into hydrogen peroxide (H_2_O_2_) [17]. H_2_O_2_ metabolism operates via the antioxidant enzyme network, which protects cells from oxidative damage and includes catalase (CAT), glutathione peroxidase 1 (GPx1), and thioredoxins. H_2_O_2_ can subsequently react with redox-active metals through Fenton chemistry, generating hydroxyl radicals that induce DNA, protein, and lipid oxidative damage [18]. Cancer cells have impaired tolerance for H_2_O_2_ when compared to non-malignant cells, including potential deficiencies in hydroperoxide metabolism enzymes, such as CAT, and reduced hydrogen peroxide removal rates [19]. These characteristics render cancer cells more susceptible to oxidative damage, presenting a vulnerability that can be exploited using targeted therapeutic strategies that generate high fluxes of H_2_O_2,_ such as high-dose intravenous vitamin C and SOD mimetics [20,21,22]. 

Removing O_2_^•−^ through SOD activity, particularly mitochondrial SOD (MnSOD), promotes wound healing via maintaining fibroblast integrity, enhancing angiogenesis in endothelial cells, and preventing oxygen toxicity that results from the respiratory burst during wound healing and wound exposure to atmospheric oxygen [23,24,25]. Previous investigations of SOD mimetics as wound-healing agents demonstrated that they may accelerate wound healing through downregulating TGF-β and TNF, suppressing inflammation, and promoting angiogenesis [23,26,27]. In contrast, increasing SOD activity using recombinant SOD, SOD overexpression, or SOD mimetics was shown to enhance radiation responses in a wide array of tumor cells, including fibrosarcoma, squamous cell carcinoma, brain, and breast cancer [28,29,30]. Thus, SOD dismutase mimetics may promote wound healing and act as a radioprotectant in non-malignant cells while simultaneously radiosensitizing cancer cells. 

**Scheme 1:**
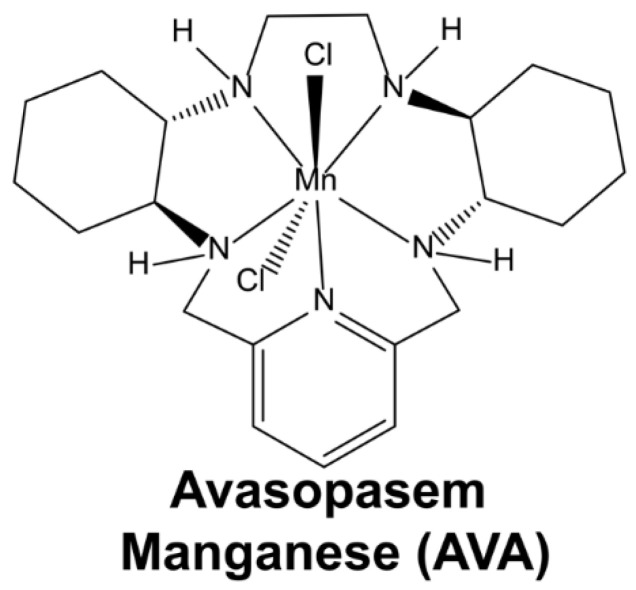
The structure of avasopasem manganese (AVA or GC4419).

One of the earliest dismutase mimetics was synthesized and characterized in the early 1990s [31]. In the ensuing decades, dismutase mimetics have been shown to protect against radiation damage in skin, soft tissue, GI mucosa, liver, brain, and lung tissues [32,33,34,35]. Avasopasem manganese (AVA or GC4419) (Scheme 1) has resulted in an enhanced bioavailability and a higher specificity to O_2_^•−^ relative to other SOD mimetics; furthermore, its catalytic rate constant of 2 × 10^7^ M^−1^ s^−1^ adequately mimics the native enzyme [35]. 

AVA significantly reduced the incidence of radiation-induced oral mucositis in head and neck cancer patients [36,37]. AVA also acts as a potent radiosensitizer in pancreatic, head-and-neck, and non-small cell lung cancers in murine models, and also markedly increases tumor response and survival in pancreatic cancer patients treated with RT [38,39,40]. Additionally, AVA attenuated cisplatin-induced renal injury in head-and-neck cancer patients [41,42]. 

The goal of this study is to explore the potential of utilizing AVA as a selective agent to enhance STS radiosensitivity and promote wound healing in normal tissues. 

## 2. Materials and Methods

### 2.1. Cell Culture, Media, and Culture Conditions

All cell lines used in this study, including SW872 (ATCC: HTB-92, liposarcoma), SKLMS1 (ATCC: HTB-88, leiomyosarcoma), and HT1080 (ATCC: CCL-121, fibrosarcoma) were obtained from the American Type Culture Collection (ATCC). The malignant peripheral nerve sheath tumor (MPNST) cell line S462 was a generous gift from Dr. Rebecca Dodd at the University of Iowa. Normal dermal fibroblasts (NDFs) were obtained from Lonza (CC-2511). All cell lines except S462 were maintained and passaged in DMEM with 10% fetal bovine serum (FBS). S462 cells were maintained and passaged in DMEM with 10% FBS, 1% Pen/strep, and 2% L-glutamine. Cells were passaged using 0.25% trypsin/EDTA. All experiments were conducted at 4% O_2_, 5% CO_2_, and 37ºC. Exponentially growing cultures of NDFs from passages 3 to 7 were used for all experiments (passage 1 was defined as when the cells were received from Lonza, Bend, OR, USA). All treatments were added to cells at a 70% confluency. 

### 2.2. In Vitro Radiation Treatment

Cells were irradiated with 2–4 Gy in the Radiation and Free Radical Research Core laboratory at the University of Iowa using a ^137^Cs source. Plates were placed in the irradiation field at room temperature and room oxygen, exposed to radiation, and then used in colony formation assays or comet assays. 

### 2.3. Animals and In Vivo Radiation Treatment

**Scheme 2:**
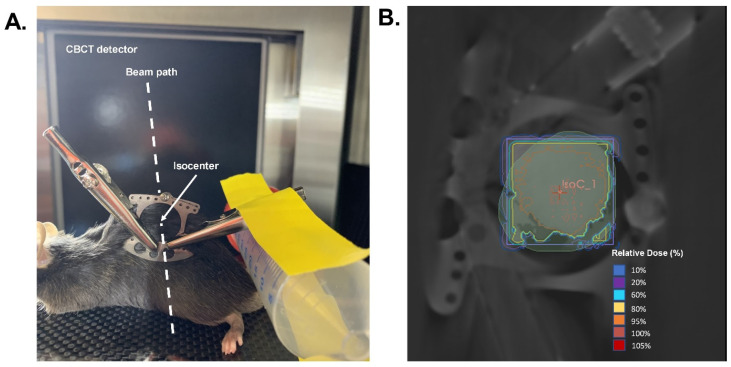
Would healing radiation treatment design. (**A**). Demonstration of radiation delivery set-up and animal positioning. (**B**). Representative radiation dose distribution. Isodose curves were generated based on a cone-beam CT image with the central field contoured and an isocenter placed in the middle of the wound field as shown in A. Mice were given a prescribed dose of 15 Gy delivered in two parallel opposed beams (90°/270°) with 50% beam weighting.

Forty 8–12-week-old male C57BL/6 mice were purchased from Envigo (Indianapolis, IN, USA). All animals were housed at the University of Iowa animal facility in a temperature-controlled room with 12 h light and 12 h dark cycles, and were maintained on a standard chow diet and water ad libitum throughout the experiment. All procedures were approved by the University of Iowa Institutional Animal Care & Use Committee under protocol #1122458. Forty-eight hours prior to radiation, animals were anesthetized using a ketamine/xylazine mixture, and a hair clipper was used to shave the back of each mouse. Metal clips were used to lift and keep the exposed skin extended from the back during radiation. The exposed skin was irradiated with 15 Gy (EQD2 = 54 Gy) with parallel opposed fields using X-Strahl Small Animal Radiation Research Platform (SARRP) at the University of Iowa Small Animal Imaging Core (Scheme 2). Thirty days following radiation, mice were anesthetized with the ketamine/xylazine mixture, the dorsal skin was tented, and two bilateral full-thickness wounds were created using a 5 mm biopsy punch (initial wound area = 20 mm^2^). Following wounding, animals were housed individually and monitored prospectively for any wounding complications, and wound closure was monitored for ten days using calipers for measurement. Ten days post-wounding (Day 40), pictures were taken of the wounds, and the percentage of the wound that was open was calculated as follows:

$$\%\;Wound\;Open=\frac{Wound\;area\;on\;Day\;40}{Initial\;wound\;area}\times 100$$

At the end of the study, the mice were euthanized using 100% CO_2_ gas, and death was confirmed via cervical dislocation. Skin samples were collected for further analyses. 

### 2.4. Tissue Staining

Following euthanasia, skin samples were harvested and fixed in 10% neutral buffered formalin. Samples were then processed, embedded, sectioned, and stained with hematoxylin and eosin (HE) and Trichrome at the University of Iowa Comparative Pathology Laboratory. A blinded board-certified pathologist analyzed the stained tissues. HE staining is represented as % Fibrosis/Scarring, describing the % of the skin occupied by fibrosis or scarring. Trichrome was used to confirm fibrosis. 

### 2.5. Preparation and Administration of Avasopasem Manganese (AVA)

AVA (MW = 483) was provided by Galera Therapeutics Inc. For all in vitro experiments, AVA was dissolved in a 10 mM sodium bicarbonate solution, pH 7.1–7.4. Cells were treated 24 h prior to radiation with 5 µM AVA [43,44,45]. For the colony formation assay, 5 µM AVA was also added to the cloning dishes. For in vivo studies, a stock solution of AVA was prepared in saline with 10 mM sodium bicarbonate. Animals received 10 mg kg^−1^ AVA IP daily, starting on the day of radiation (one hour before), and lasting for the entire duration of the study (40 days) [42,46,47]. 

### 2.6. Colony Formation Assay

To determine the effect of RT and AVA on the reproductive integrity in STSs, 50–100 K cells were plated and were allowed 24 h to attach. Cells were treated with 5 µM AVA or 1 mM buthionine sulfoximine (BSO) for 24 h and exposed to 2 Gy of ionizing radiation. Cells from treated and control dishes were harvested using 0.25% Trypsin/EDTA. Following the inactivation of trypsin with 10% FBS-containing media, cells were counted using a Coulter Counter and plated at various densities in 6-well plates in complete media for 7–10 days. During the assay, NDFs were grown on a feeder layer of Chinese hamster fibroblasts (lethally irradiated with 30 Gy) as previously described [48]. Coomassie Blue dye was used to stain cells to visualize colonies. Colonies with at least 50 cells were counted, and clonogenic cell survival was determined as previously described [48,49]. The normalized survival fraction (NSF) was determined as the surviving fraction of any given clonogenic plate from a treatment group divided by the average surviving fraction of the control (untreated) plates within a given experiment.

### 2.7. Migration Assay

Exponentially growing NDF and STS cells were seeded in full media into 24-well Corning Fluoro-Blok Cell Culture Inserts (Fisher Scientific 8.0 µm pore size, #351152, Waltham, MA, USA). For the RT group, cells were irradiated with 4 Gy before being placed into trans-well chambers. The plates were incubated for 24 h at 37 °C, 4% oxygen. Cells were pre-treated for 24 h with 5 µM AVA before RT. Following 24 h of plating, media was aspirated from the top chamber, and the non-invading cells were removed from the upper chamber using a cotton swab. Invading cells (on the bottom layer of the membrane) were stained by placing the trans-well chambers into 0.5 mg/mL of a Thiazolyl Blue Tetrazolium Bromide (MTT) solution (Sigma-Aldrich #M5655, Saint Louis, MO, USA) in media without FBS or phenol red. Inserts were incubated at 37 °C for 1 h before membranes were removed and incubated in 150 μL of DMSO. The DMSO solution was transferred to a 96-well plate, and the absorption at 550 nm was measured using a plate reader. Data were normalized to the untreated control.

### 2.8. Alkaline Comet Assay

Alkaline Comet assays were carried out using the R&D CometAssay Electrophoresis Starter Kit (#4250-050-ESK, Minneapolis, MN, USA) following the manufacturer’s instructions, with slight modifications as previously described [49]. Briefly, cells were placed on 2-well comet slides following resuspension in agarose and allowed to dry in the dark at 4 °C for 10 min. Lysis was carried out for 45 min at 4 °C followed by a 20 min incubation in alkaline buffer at room temperature. Electrophoresis was carried out at 21 V for 30 min. Subsequently, slides were washed twice in water, once in 70% ethanol, and then were left to dry at 37 °C for 15 min. Furthermore, 1X SYBR Gold (#S11494, ThermoFisher, Waltham, MA, USA) stain was added to the slides for 30 min at room temperature in the dark. After a brief rinse in water, slides were allowed to dry at 37 °C for 10 min. Fluorescent microscope images were analyzed using the autoanalyzer software CometScore 2.0.0.38 to obtain percent tail DNA (http://rexhoover.com/index.php?id=cometscore, accessed on 21 December 2021).

### 2.9. Western Blotting

Here, 30 ug of protein from cell lysates homogenized in RIPA buffer was loaded on a 4–20% gel (BIORAD, #4561095, Hercules, CA, USA) and run at 95V for 60 min. Protein was transferred to a PVDF membrane (BIORAD, #1620264, Hercules, CA, USA) at 100V for 1 h at 4 °C. The blot was blocked with 5% non-fat dry milk in TBST for 1 h at room temperature and was subsequently incubated with antibodies against catalase (1:1000, Cell Signaling #14097, Danvers, MA, USA), glutathione peroxidase 1 (1:1000, Cell Signaling, #3206), and GAPDH (1:1000, Cell Signaling, #2118, Danvers, MA, USA) overnight at 4 °C. Rabbit (1:10,000, Cell Signaling, #7074, Danvers, MA, USA) or mouse (1:10,000, BD Transduction Laboratories, #M15345, Franklin Lakes, NJ, USA) secondary antibodies were added to the blot for 1 h at room temperature. Following incubation with the secondary antibody, SuperSignal™ West Pico PLUS chemiluminescence substrate (ThermoFisher, #34580, Waltham, MA, USA) was added to the blot for 5 min and an X-ray film (Research Products International, #248300, Mt Prospect, IL, USA) was used to obtain images. Images were quantified using ImageJ.1.53k.

### 2.10. Catalase Activity

Catalase activity was determined by detecting the disappearance of H_2_O_2_ at 240 nm, ε240 = 39.4 M^−1^ cm^−1^ using Abei’s method via UV-Vis spectroscopy [50]. 55.6 mM potassium phosphate buffer (pH 7.0) was used as the working buffer for the assay. Additionally, 30 mM of H_2_O_2_ (Sigma-Aldrich, H1009, Saint Louis, MO, USA) was used to initiate the catalase reaction to achieve a final H_2_O_2_ concentration of 10 mM in the assay cuvette. Immediately, the absorbance of H_2_O_2_ was measured upon the start of the reaction and for 120 s (s) at 10 s intervals. kU of catalase activity was determined using the natural log of the rate of H_2_O_2_ disappearance. Sample protein was measured using the DC Protein Assay Kit (BIORAD, #5000111, Hercules, CA, USA) and was used to obtain normalized catalase activity. 

### 2.11. Glutathione Peroxidase Activity

Lawrence and Burk’s method was used to determine glutathione peroxidase 1 (GPx1) spectrophotometrically [51]. Samples and standards were measured in a buffer containing reduced glutathione (Sigma-Aldrich, G4251, Saint Louis, MO, USA) (1 mM), glutathione reductase (Sigma-Aldrich, G3664, Saint Louis, MO, USA) (1 EU/mL), and NADPH (Sigma-Aldrich, 10107824001) (0.2 mM). The activity was determined by measuring the disappearance of NADPH at 340 nm following the addition of H_2_O_2_ (final concentration = 0.25 mM). Furthermore, 1 μmole of NAPDH oxidized/min was used to define 1 unit of GPx1. The activity was then normalized to protein content in the sample as measured using the DC Protein Assay Kit.

### 2.12. Statistical Analysis

One-way or two-way ANOVA with Tukey’s multiple comparison test were used to analyze colony formation assays, DNA damage data, migration data, catalase activity, GPx1 activity, and wound closure data. Student’s *t*-tests with Welch’s correction were used to analyze histological data (after performing a conservative outlier test, ROUT (Q = 0.1%)). Correlation analyses were completed using simple linear regression. *p* ≤ 0.05 was considered statistically significant. GraphPad Prism 9 software was used for all statistical analyses.

## 3. Results

### 3.1. AVA Selectively Sensitizes STS Cells to Radiation Therapy, Reduces STS Migration, and Protects Normal Cells against Single-Strand DNA Breaks

**Figure 1:**
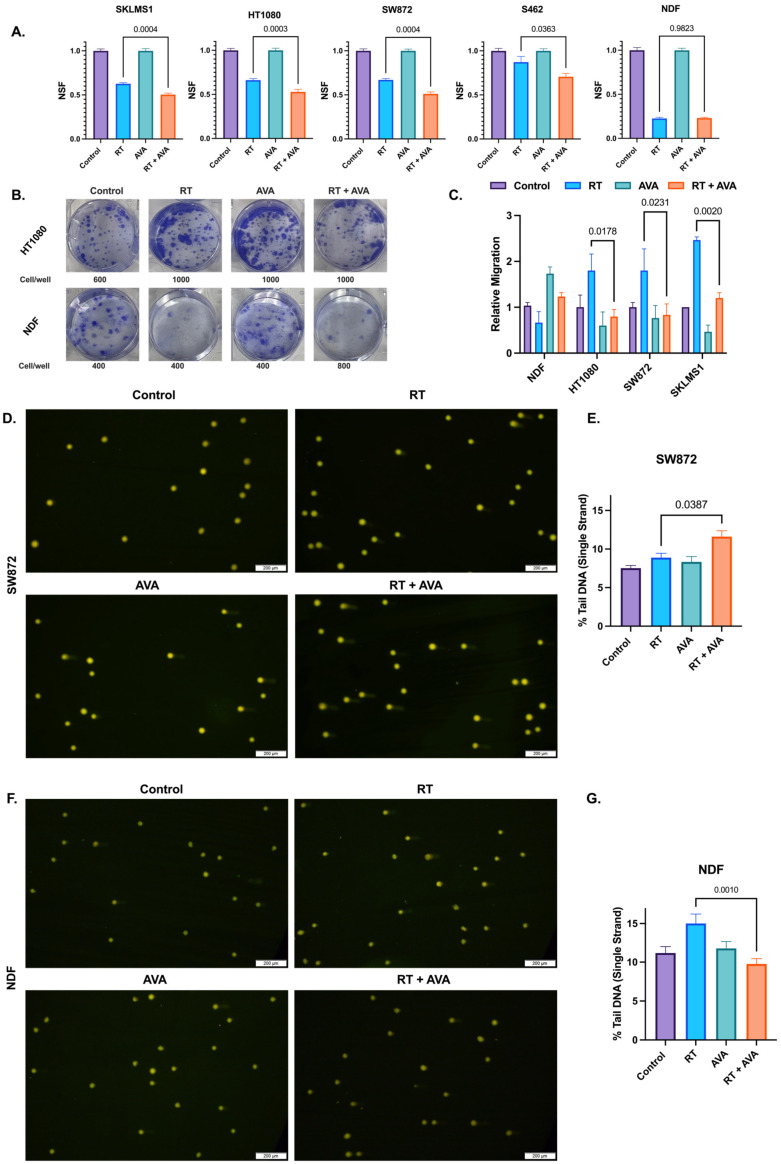
AVA selectively sensitizes STS cells to radiation therapy and protects normal cells against DNA damage in vitro. (**A**). Clonogenic survival in SKLMS1 leiomyosarcoma cells, HT1080 fibrosarcoma cells, SW872 liposarcoma cells, S462 MPNST, and NDFs. Data shows untreated controls, cells treated with RT (2 Gy), AVA, or RT + AVA (5 µM). (**B**). Representative images of clonogenic cell survival following treatment with RT + AVA between HT1080 (top) and NDFs (bottom). (**C**). Relative migration of NDFs, HT1080 fibrosarcoma, SW872 liposarcoma, and SKLMS1 leiomyosarcoma cells following treatment. (**D**). Representative images of alkaline comets in SW872 following treatment (2 Gy RT, 5 µM AVA). (**E**). Percent tail DNA (single strand) in SW872 cells following treatment. (**F**). Representative images of alkaline comets in NDFs following treatment (2 Gy RT, 5 µM AVA) (**G**). Percent tail DNA (single strand) in NDFs following treatment. N = 3 for each experiment. Error bars represent standard error of the mean (SEM).

The efficacy of AVA and RT on STSs and NDFs was assessed using a colony formation assay, a classic radiobiology tool used in the evaluation of cellular responses to radiation therapy with and without other cytotoxic agents [52,53,54]. AVA, when combined with RT, significantly reduced clonogenic survival in fibrosarcoma (*p* = 0.0003), liposarcoma (*p* = 0.0004), leiomyosarcoma (*p* = 0.0004), and MPNST (*p* = 0.0363) (Figure 1A,B), and this effect was observed at up to 4 Gy in three of the four cell lines (Figure S1A–D). No significant differences were observed in NDFs between RT alone and the RT + AVA group (*p* = 0.982) (Figure 1A,B and Figure S1E). RT appeared to increase migration for all three STS cell lines, while possibly decreasing it for NDFs. AVA significantly reversed the increase in migration following RT in fibrosarcoma (*p* = 0.0178), liposarcoma (*p* = 0.0231), and leiomyosarcoma (*p* = 0.002) (Figure 1C). While not statistically significant, AVA also appeared to rescue normal cell migration following RT when compared to RT alone (*p* = 0.3) (Figure 1C). 

To further evaluate the effects of AVA on STSs and NDFs, we investigated DNA damage. DNA damage is a primary mechanism of the action of RT [13]. H_2_O_2_-generating treatments such as AVA can form hydroxyl (^.^OH) radicals generated through Fenton chemistry due to the reaction between H_2_O_2_ and ferrous iron, yielding in site-specific DNA breaks [18,55]. Thus, DNA damage was assessed in STSs and NDFs following RT and AVA using alkaline comet assays to detect single-strand DNA breaks. Consistent with the colony formation assay, AVA in combination with RT significantly increased single-strand DNA breaks in SW872 (liposarcoma) when compared to RT alone (*p* = 0.0387) (Figure 1D,E), aligning with enhanced clonogenic death. In contrast, AVA effectively protected NDFs against RT-induced DNA damage with a significant decrease in single-strand breaks between RT and the RT + AVA group (*p* = 0.001) (Figure 1F,G), consistent with clonogenic survival rates. Taken together, these data show that AVA demonstrates selective toxicity in STSs when compared to non-malignant cells, and also protects non-malignant cells from RT-induced DNA damage. 

### 3.2. Alterations in Hydrogen Peroxide Metabolism May Potentiate the Selective Toxicity of AVA in STSs

**Figure 2:**
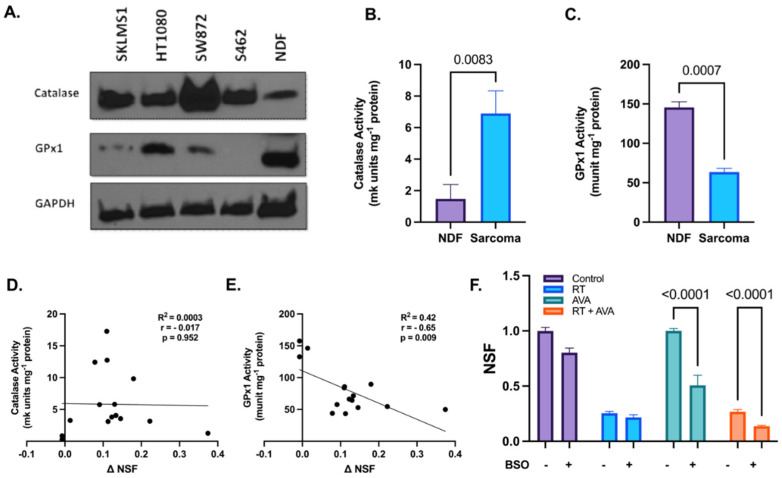
Alterations in hydrogen peroxide metabolism may potentiate the selective toxicity of AVA in STSs. (**A**). Immunoblotting of CAT and GPx1 in various STS cell lines and NDFs, GAPDH was used as a loading control (*n* = 1). (**B**). CAT activity (mk units mg^−1^ protein) in NDF and STS cells (SKLMS1, HT1080, SW872, and S462). (**C**). GPx1 activity (munit mg^−1^ protein) in NDF and sarcoma cells (SKLMS1, HT1080, SW872, and S462). (**D**). Correlation between catalase activity (mk units mg^−1^ protein) and ΔNSF (ΔNSF = NSF_RT_ − NSF_RT+AVA_) in STSs and NDFs. (**E**). Correlation between GPx1 activity (munits mg^−1^ protein) and ΔNSF in STSs and NDFs. (**F**). Clonogenic survival of NDFs treated with RT + AVA with and without BSO-induced glutathione depletion. *N* = 3. Error bars represent standard error of the mean (SEM).

H_2_O_2_ metabolism plays a crucial role in combating cellular oxidative damage due to excessive ROS and aids in maintaining cellular homeostasis [56]. Since RT has been shown to increase steady-state levels of H_2_O_2_ [57], we hypothesized that the intracellular clearance of H_2_O_2_ was impaired in STSs relative to normal cells, resulting in increased sensitivity to the excess H_2_O_2_ generated by AVA. To test this hypothesis, the immunoreactive protein levels of two pivotal enzymes involved in H_2_O_2_ clearance, i.e., CAT and GPx1 were assessed at the baseline in each of the four STS lines and NDFs [58]. Western blotting showed increased protein levels of CAT, but decreased levels of GPx1 in STS cell lines relative to NDFs (Figure 2A,B). In conjunction with protein levels, changes in the activity of CAT and GPx1 were assessed using UV spectrophotometry. Results indicate a significant increase in CAT activity in STS cells when compared to NDFs (6.9 and 1.5 mk units mg^−1^ protein, respectively, *p* = 0.0083) (Figure 2C and Figure S2A). In contrast, GPx1 activity was significantly increased in NDF cells when compared to STS cells (146 and 63.6 munit mg-1 protein, respectively, *p* = 0.0007) (Figure 2D and Figure S2B). Furthermore, CAT activity showed no significant correlation with the change in normalized surviving fractions (NSFs) (ΔNSF = NSF_RT_ − NSF_RT+AVA_) (r = −0.017, *p* = 0.952) (Figure 2E), while GPx1 activity significantly negatively correlated with ΔNSF (r = −0.65, *p* = 0.009) (Figure 2F). To further investigate the role of GPx1 in the selective toxicity of AVA, we tested whether glutathione depletion using BSO (an inhibitor of glutamate–cysteine ligase, the rate-limiting enzyme in the synthesis of glutathione) would increase the toxicity of AVA in NDFs. Treatment with BSO significantly sensitized NDFs to both AVA alone (*p* < 0.0001) and RT + AVA (*p* < 0.0001) (Figure 2F). These data suggest a potential alteration in H_2_O_2_ metabolism, particularly GPx1, that may render the STS cells vulnerable to high fluxes of H_2_O_2_ due to impairments in the detoxification process. 

### 3.3. AVA Accelerates IR-Induced Wound Healing in a Murine Model

**Figure 3:**
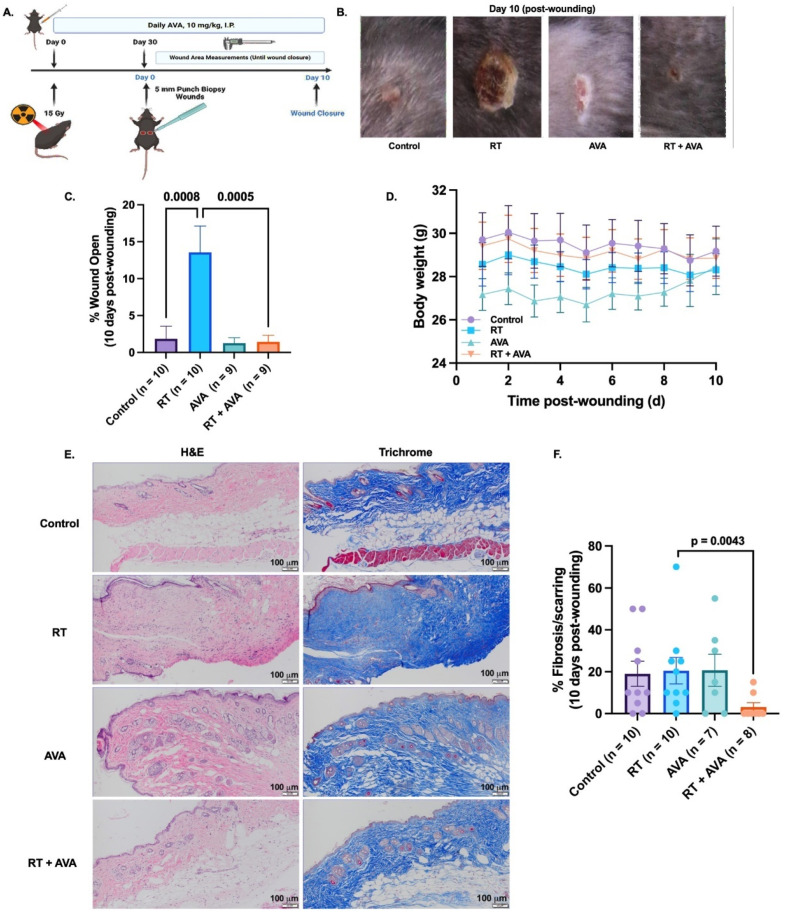
AVA accelerates RT-induced wound healing in a murine model. (**A**). Schematic of experimental design and mouse model of RT-induced wound healing impairment. (**B**). Representative images of wounds 10 days post wounding. (**C**). Percent wound closure on day 10 post wounding. (**D**). Body weights (gms) over time (days) during the 10-day wound monitoring period. (**E**). Representative HE (top) and trichrome (bottom) images of mouse skin from untreated mice and mice treated with RT, AVA, and RT + AVA, 10 days post wounding 100X magnification, scale = 20 µm. (**F**). Quantification of percent fibrosis (HE) in mouse skin harvested 10 days post wounding. *p* ≤ 0.05 is statistically significant. Error bars represent standard error of the mean (SEM).

We utilized a murine model of irradiated skin (Scheme 1, Figure 3A) to test the effect of AVA on RT-delayed wound closure. RT significantly decelerated the rate of wound closure over 10 days post wounding, compared to the unirradiated control (% wound open = 13.6% with RT versus 1.8% in control, *p* = 0.0008) (Figure 3B,C). Interestingly, AVA significantly restored wound healing in RT-treated animals when compared to RT alone (% wound open = 1.4% with RT + AVA versus 13.6% with RT alone, *p* = 0.0005) (Figure 3B,C). No significant differences were observed between control wounds and those treated with RT + AVA (*p* = 0.999) or with AVA alone (*p* = 0.997). These striking in vivo observations demonstrate a significant potential for AVA as a wound-healing agent with nRT. No significant changes in body weight were observed during recovery from wounding (Figure 3D). Furthermore, the histological evaluation of skin collected at the end of the study was performed to evaluate scarring/fibrosis-related changes in the skin following treatment with RT and AVA. There were no differences between the control and RT samples regarding the percent fibrosis (Figure 3E,F), suggesting that, due to the short timeline of the study (40 days post radiation), a chronic wound-related fibrosis effect could not be detected [59,60]. Interestingly, RT + AVA samples showed a significant reduction in %fibrosis (*p* = 0.0043) (Figure 3E,F), suggesting a potential anti-fibrosis effect of AVA that warrants investigation in a chronic radiation wound model. 

## 4. Discussion

Soft tissue sarcomas (STSs) are rare, heterogeneous, locally aggressive, and highly metastatic tumors known for poor patient outcomes. STS treatment options include combinations of RT, surgery, and chemotherapy. Furthermore, current treatments may cause dose-limiting toxicities that also impact the quality of life post therapy. Although neoadjuvant RT (nRT) combined with limb-sparing surgery is currently the most effective tumor control strategy, approximately 30% of patients treated with this approach develop wound healing complications post surgery, including infections, a significant need for dressing changes, wound dehiscence, and necrosis. An emerging strategy to combat wound complications post nRT is the administration of dismutase mimetics concurrent with RT. AVA is a small molecule selective dismutase mimetic that has recently shown high efficacy in mitigating radiation-induced oral mucositis and cisplatin-induced kidney injury in patients with head and neck cancer [33,36,37]. Moreover, AVA enhanced radiation response and increased survival in patients with pancreatic cancer [38,40]. We investigated the dual role of AVA as a radiosensitizer in STSs and a radioprotector in normal cells and murine skin, including in a model of wound healing post RT and surgery. 

When combined with RT in vitro, AVA significantly reduced clonogenic survival in several STS cell lines, including leiomyosarcoma, fibrosarcoma, liposarcoma, and MPNST. In contrast, no significant change in colony formation was observed in normal dermal fibroblast (NDF) cells, suggesting a selective role for AVA in increasing the sensitivity of STSs to RT. Furthermore, given the importance of cell migration in cancer and wound healing, we also evaluated the migration of STS and NDF cells following treatment with RT and AVA. RT appeared to reduce NDF migration, but increase STS migration, the latter being an effect that has been seen with other cancers. AVA reversed this and significantly reduced STS migration following RT, as compared to fibroblasts where it appeared to promote migration. Although the increase in NDF migration promoted by AVA did not meet statistical significance, it may be an avenue that warrants further investigation due to the importance of fibroblast migration during wound healing [61]. Furthermore, STS cells demonstrated increased levels of DNA damage following treatment with IR + AVA when compared with RT alone, which contrasted with normal fibroblasts, where there was a significant AVA reduction in RT-induced DNA damage. DNA damage data suggest that AVA exacerbates DNA damage in STSs in combination with RT, leading to the enhanced cell killing observed in the clonogenic assay [45,62]. In total, our findings are consistent with previous works showing that AVA protects normal cells from cancer therapy and aging-associated damage [48]. Moreover, the radio-sensitizing effects of AVA in STSs were consistent with those previously shown in pancreatic, head-and-neck, colorectal, and non-small cell lung cancer, thus warranting further investigation in a murine model of STSs [38,39,45,47,63]. 

To gain mechanistic insight into the selective toxicity of AVA, the immunoreactive protein levels and enzymatic activities of two H_2_O_2_ metabolism enzymes, CAT and GPx1, were investigated in STS cells as compared to normal cells. STS cells showed increased CAT, but decreased GPx1 protein expression relative to NDF cells. To gain a more robust insight into these differences, we evaluated the enzymatic activity of CAT and GPx1 in NDF and STS cells, as the enzymatic process of H_2_O_2_ removal is more physiologically relevant as intracellular H_2_O_2_ content is regulated via the active enzymatic actions of CAT and GPx1. Consistent with immunoreactive protein, STS cells showed significantly elevated CAT activity and significantly reduced GPx1 activity when compared to their NDF counterparts. Furthermore, higher GPx1 activity significantly correlated with less radio-sensitization (measured as Δ NSF) with AVA, suggesting a potential role of GPx1 in AVA selective toxicity as CAT did not demonstrate any significant correlation with AVA radio-sensitization. Although the CAT activity was significantly higher in STSs, our data suggest that STSs may depend upon glutathione metabolism and GPx1 for intracellular H_2_O_2_ detoxification when treated with RT + AVA. Moreover, it has been shown that elevated tumor CAT may be associated with CAT translocation from the peroxisome to the cell membrane, and thus its activity and protein levels may not reflect intracellular H_2_O_2_ clearance [64]. To further test the potential role of GPx1 in AVA selectivity, BSO was used to inhibit GPx1 activity in NDFs through glutathione depletion. As hypothesized, BSO significantly sensitized NDFs to AVA toxicity, both alone and combined with RT, thus supporting the mechanistic role of GPx1 in AVA selectivity, similarly to a previous study reporting that GPx1 inhibition sensitized lung cells to AVA [39]. Additionally, this finding opens the door to potentially investigating BSO combined with AVA as a means to overcome chemo- and radioresistance in STSs with high GPx1/glutathione, especially with a clinical trial reporting potentially enhanced responses to melphalan in neuroblastomas with elevated glutathione [65]. Taken together, these data suggest that relatively low levels of GPx1 may mediate selective AVA toxicity in STSs and warrant further investigation.

Next, we tested the efficacy of AVA on wound closure in a murine model treated with nRT. Mice were treated with 15 Gy/fraction targeted to isolated skin, and AVA (10 mg kg^−1^, IP) was administered daily for the duration of the study. Wounds were created in the irradiated skin 30 days following radiation to mimic the clinical sequence of nRT and surgery. Radiation significantly delayed wound closure in the RT group compared to the untreated and AVA-only controls, consistent with the literature on radiation wound complications [12,66,67,68]. Combining AVA with RT nearly completely reversed the RT-induced delay in wound closure. These data show that AVA can prevent RT-induced wound healing impairment. The histological evaluation of the skin showed no difference in fibrosis/scarring between untreated controls and RT or AVA, likely due to the acute nature of the study. However, interestingly, RT + AVA samples showed a significant reduction in scarring/fibrosis when compared to RT alone, suggesting a potential antifibrotic role of AVA that should be further investigated in a chronic setting. While this study provided preliminary evidence on AVA as a wound-healing agent, multiple factors must be addressed in future studies, including dosing, inflammation, and fibrosis. The effects of AVA on wound healing require evaluation at the molecular and cellular level, including the assessment of immune infiltrates, fibroblasts, and keratinocytes, and the expression of inflammatory markers and proteins associated with wound healing. These future studies are necessary, especially with recent findings in Cisplatin-induced kidney injury, where a key mechanism of AVA was its anti-inflammatory function that significantly reduced protein levels of TNF-α, one of the regulators of wound healing, inflammation, and fibrosis [42,69,70].

In conclusion, this study showed that AVA has a dual function as a potent radiosensitizer for STS cells and a radioprotective agent for NDFs, and this selective effect is potentially driven by relative GPx1 activity. Furthermore, in vivo, AVA demonstrated promising preliminary results in promoting wound healing following radiation through its radioprotective effects on normal skin. 

There are certain limitations to the models utilized and findings reported in this study that need to be addressed in future studies. One key limitation is the connection between H_2_O_2_ metabolism and the selectivity of AVA in STS versus NDF cells; addressing this limitation requires glutathione depletion in STSs in addition to genetic manipulations of CAT and GPx1 in order to elucidate their respective effects on AVA toxicity in relation to the H_2_O_2_ content, as measured using redox-sensitive probes such as H2DCFDA [71]. This would inform whether the mechanism of AVA (and other SOD mimetics) in STSs is similar to the previously reported H_2_O_2_-mediated oxidative damage or if there is a novel mechanism that is unique to STSs [20,39,41,42,43,46,48,72,73]. 

Another limitation is related to the murine wound healing model. While this model provided preliminary insights regarding how AVA may protect the skin and mitigate RT-associated wound healing complications, extrapolating these findings to human STS patients needs caution. Radiation volumes when treating a STS are significantly larger and often include skeletal muscle, cartilage, and other connective tissues. These tissues are of great significance, particularly in the extremities, as wound healing complications are often accompanied by muscle and joint stiffness [10,11]. 

However, these data are of great translational significance due to the critical need for novel therapeutic approaches to enhance RT efficacy in STSs and minimize RT-associated normal tissue toxicities. These findings warrant further pre-clinical investigations to fill in the knowledge gaps in the redox biology of STSs, and to guide future clinical trials that may improve therapeutic outcomes in STS patients receiving nRT, preserving their quality of life by minimizing wound healing complications.

## Data Availability

Data is available upon request.

## Supplementary Materials

The following supporting information can be downloaded at: https://www.mdpi.com/article/10.3390/antiox13050587/s1.
