# Supplementary material for: Superoxide Dismutase Mimetic Avasopasem Manganese Enhances Radiation Therapy Effectiveness in Soft Tissue Sarcomas and Accelerates Wound Healing
Source: Antioxidants (Basel). 2024 May 10;13(5):587. doi: 10.3390/antiox13050587 (PMC11117842; doi:10.3390/antiox13050587)
Supplement: Supplementary file 1 [file antioxidants-13-00587-s001.zip › antioxidants-2969416-supplementary.pdf]

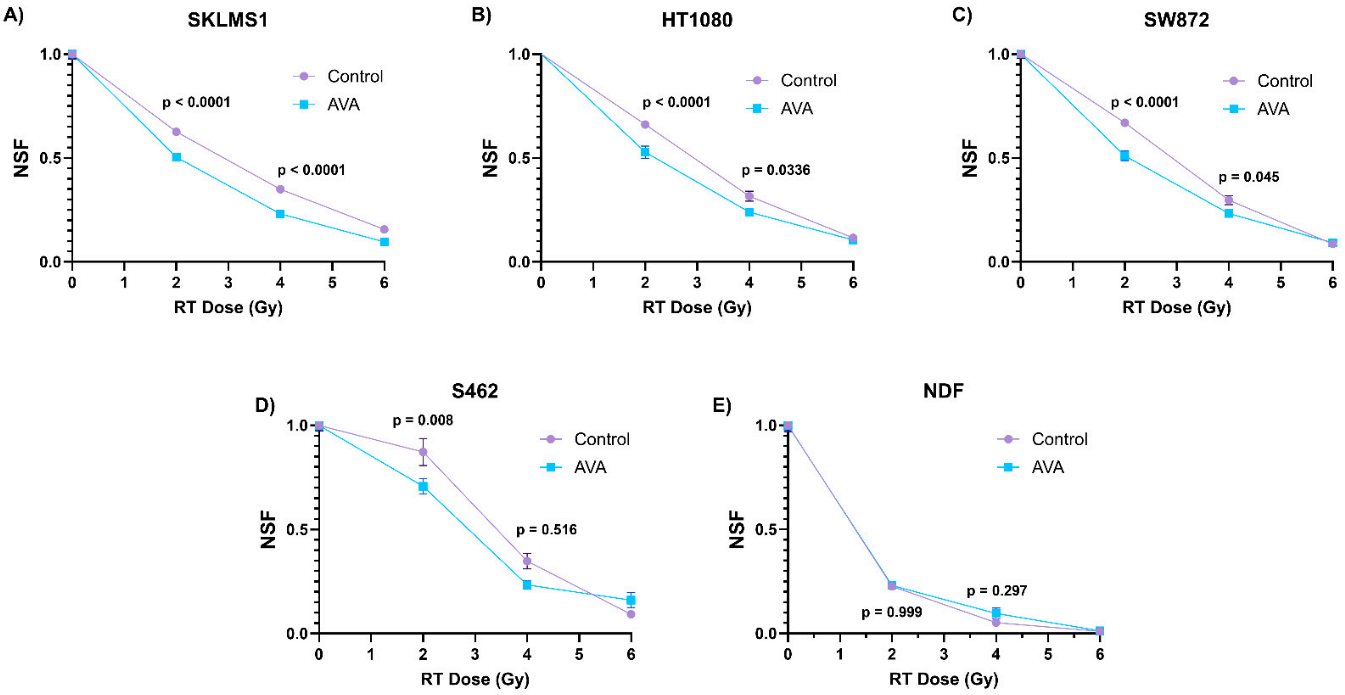

Figure S1. Normalized survival fraction (NSF) in SKLMS1 leiomyosarcoma **(A)**, HT1080 fibrosarcoma cells **(B)**, SW872 liposarcoma cells **(C)**, S462 MPNST cells **(D)**, and Normal dermal fibroblasts (NDFs) **(E)** treated with 0, 2, 4, and 6 Gy or radiation ± AVA. N = 3. $p \leq 0.05$ is statistically significant.

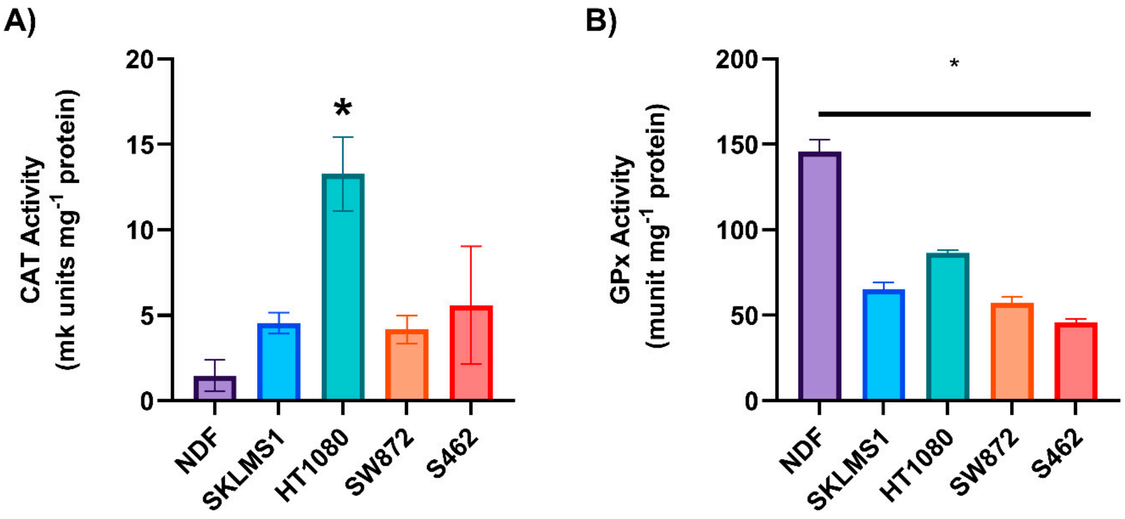

Figure S2. Catalase (CAT) and glutathione peroxidase 1 (GPx1) activity in sarcomas and NDFs. **(A)** CAT activity in NDFs, SKLMS1, HT1080, SW872, and S462 (mk units mg^{-1} protein). **(B)** GPx1 activity in NDFs, SKLMS1, HT1080, SW872, and S462 (munit mg^{-1} protein). N = 3. * $p \geq 0.05$, statistically significant.
